# Expression of Human Immunodeficiency Virus type 1 (HIV-1) coat protein genes in plants using cotton leaf curl Multan betasatellite-based vector

**DOI:** 10.1371/journal.pone.0190403

**Published:** 2018-01-05

**Authors:** Elham Ataie Kachoie, Seyed Ali Akbar Behjatnia, Sara Kharazmi

**Affiliations:** 1 Institute of Biotechnology, Shiraz University, Shiraz, Iran; 2 Plant Virology Research Center, College of Agriculture, Shiraz University, Shiraz, Iran; Indiana University, UNITED STATES

## Abstract

It has already been demonstrated that a betasatellite associated with cotton leaf curl Multan virus (CLCuMB) can be used as a plant and animal gene delivery vector to plants. To examine the ability of CLCuMB as a tool to transfer coat protein genes of HIV-1 to plants, two recombinant CLCuMB constructs in which the CLCuMB βC1 ORF was replaced with two HIV-1 genes fractions including a 696 bp DNA fragment related to the HIV-1 *p24* gene and a 1501 bp DNA fragment related to the HIV-1 *gag* gene were constructed. *Gag* is the HIV-1 coat protein gene and *p24* is a component of the particle capsid. Gag and p24 are used for vaccine production. Recombinant constructs were inoculated to *Nicotiana glutinosa* and *N*. *benthamiana* plants in the presence of an Iranian isolate of Tomato yellow leaf curl virus (TYLCV-[Ab]) as a helper virus. PCR analysis of inoculated plants indicated that *p24* gene was successfully replicated in inoculated plants, but the *gag* gene was not. Real-time PCR and ELISA analysis of *N*. *glutinosa* and *N*. *benthamiana* plants containing the replicative forms of recombinant construct of CLCuMB/p24 indicated that *p24* was expressed in these plants. This CLCuMB-based expression system offers the possibility of mass production of recombinant HIV-1 *p24* protein in plants.

## Introduction

Biotechnology methods using plants as an expression system offer the possibility of producing recombinant proteins for medical or veterinary applications [1, 2]. Plants have many advantages over other expression systems for producing recombinant proteins. Compared to bacteria, yeast and mammalian and insect cell cultures, plants have very low production costs, very high scale-up potential, and require low initial investments [3]. The use of plants and cell cultures to produce recombinant proteins, termed “molecular farming” [4], offers a great potential for producing high-value, cheaper, faster and widely available pharmaceuticals. In the case of viral vaccines, in particular, it has been proven that fully-assembled complex virus like particles and immunoglobulins can be made which in turn can elicit immune responses and reduce side effects following their applications [4].

Plant-derived vaccines can be produced in transgenic plants or by plant viral vectors [5]. *Agrobacterium*-mediated transient expression is another means of producing biopharmaceuticals in plants offering advantages such as easy manipulation, speed, low cost and high yield of protein productions compared to stable transgenic systems [6]. It is often difficult to generate transgenic plants with sufficient expression level. In addition, high expression of foreign proteins may be toxic to plants [7]. Foreign gene expression from viral vectors in plants is predicted to occur usually within two or three weeks after inoculation. Plant viruses are easily transmissible and could potentially be used commercially for rapid mechanical inoculation and agroinoculation to a large number of crop plants [1, 7–11], although natural transfer of plant viruses can present some problems [8].

Among the viral vectors tested, geminiviruses (family *Geminiviridae*) have been successfully used as replicons for transient gene expression [12]. Geminiviruses are small circular single-stranded (ss) DNA viruses infecting plants. Their genomes are encapsidated in twinned icosahedral particles. Based on criteria including genome organization, insect vectors, host range and genome-wide pairwise sequence identities, the family *Geminiviridae* has been recently classified into nine genera including the genus *Begomovirus* [13]. Viruses in the genus *Begomovirus* have either one or two genomic components [13]. Virus replication occurs in the nucleus of the host plant cell through double-stranded replicative intermediates by a rolling circle mechanism [14]. Geminivirus replication also occurs via a recombination-dependent mechanism [15]. The copy number and the expression levels of foreign genes that are linked to the geminiviral replicons could efficiently be enhanced as these ssDNA viruses possess multiple features in their replication process that make them potentially useful in gene amplification strategies [16, 17]. Expression of a wide range of single or multiple products in many plant families could be made possible by using the appropriate replicating vectors derived from several different geminiviruses [18]. Development of virus inducing gene silencing (VIGS) vectors derived from broad host range geminiviruses or satellites DNAs associated with begomoviruses have been already reported [10, 19–21]. Furthermore, systems using geminiviruses vectors to deliver reagents such as nucleases for plant genome engineering and to express proteins at levels useful for commercial production of vaccines and other proteins in plants have been recently developed [11, 22, 23].

In addition to the main viral genome, circular ssDNA molecules, referred to as betasatellite (DNA β), are exclusively associated with certain monopartite begomoviruses including Cotton leaf curl Multan virus (CLCuMV) [24, 25]. The betasatellite associated with CLCuMV (CLCuMD) is approximately half the size of viral genomic DNA (1351 nt in size) and encodes only a single complementary-sense open reading frame (ORF), βC1 (354 nt in size), which is a pathogenicity determinant [26]. However, it depends on a helper virus for its replication and encapsidation [26, 27]. Various studies have indicated that CLCuMB can be used as a gene delivery vector to plants. Inoculation of different plants with infectious recombinant CLCuMB constructs in which βC1 replaced with another gene resulted in silencing of homologous gene activities, indicating that the CLCuMB can be used as a strong VIGS vector [10]. A resistance strategy against geminiviruses based on the activation of an integrated barnase gene inserted into the CLCuMB DNA by the helper virus replication-associated protein (Rep) was reported previously [28]. This strategy offered the potential resistance against geminiviruses in a variety of plant species that support the replication of CLCuMB [28]. A specific insertion construct in which a human B-cell lymphoma 2 (Bcl-2) cDNA (720 bp in size) was introduced into the CLCuMB was replicated in tobacco and tomato plants in the presence of various geminiviruses. The Bcl-2 gene was expressed in these plants indicating that CLCuMB can be used as a gene delivery and expression vector of animal genes in plants [29].

Human Immunodeficiency Virus type 1 (HIV-1), a lentivirus (family *Retroviridae*), causes acquired immunodeficiency syndrome (AIDS), a chronic and debilitating disease that cannot be cured with current antiretroviral drugs [30–32]. However, new biological and gene-based therapeutics have been reached to clinics. In these therapies, the RNA interference approaches haves been used to silence the expression of target host or virus transcripts that are required for HIV-1 genome replication and viral infection [32]. The HIV-1 genes can also be used to produce recombinant vaccines against the virus [31, 33, 34]. Application of these therapeutic strategies requires the transcripts of genes and proteins involved in the production of HIV-1 antibodies. The HIV-1 genome comprises nine functional genes including *gag*-, *pol*-, *vif*-, *vpr*-, *tat*-, *rev*-, *vpu*-, *env*- and *nef*-gene that are arranged in three different reading frames. These genes encode for structural/enzymatic, regulatory and accessory proteins that are required for all processes in the viral replicative cycle [30, 32, 35]. Among these genes, *gag* is one of the most conserved HIV genes. It is initially produced as a 55 KDa precursor protein which is cleaved by a protease to the matrix, capsid, nucleocapsid, and P6 proteins following the virus budding. The protease is encoded as a part of the gag/Pol polyprotein [36, 37]. The capsid protein, known as p24, is a structural protein that makes up most of the HIV-1 viral core. *Gag* and *p24* genes are very conserved in HIV-1 genome and are currently used for vaccine production [11, 38–41]. We report here the construction and testing of a replicating gene expression vector based on CLCuMB to transcribe HIV-1 *p24* mRNA and to express HIV-1 *p24* protein in plants in the presence of an Iranian isolate of Tomato yellow leaf curl virus (TYLCV-[Ab]) as a helper virus.

## Materials and methods

### Plant material and growth conditions

Seeds of *Nicotiana glutinosa* and *N*. *benthamiana* were sown in small pots containing peat moss and sand in a 2:1 ratio, and grown in plant growth chambers at 25 ± 2°C with a 16 h light / 8 h dark regime.

### Viruses and CLCuMB infectious clones

Infectious constructs of Tomato yellow leaf curl virus (TYLCV-[Ab]) *Sac*I/*Sph*I head-to-tail 1.5 mer in pBin19 (pBin-1.5TYLCV-[Ab]) [28], CLCuMB *Kpn*I/*Sna*BI head-to-tail 1.2 mer in pBin20 (pBin-1.2β) [26], a mutant version of CLCuMB in which the βC1 gene was deleted (DNAβΔC1) in pTZ57R/T (pTZβΔC1) [28] and a head to tail partial repeat of the CLCuMB mutant (pBin-1.2βΔC1) [29], all described previously, were used in this study.

### Recombinant CLCuMB clone

**Fig 1:**
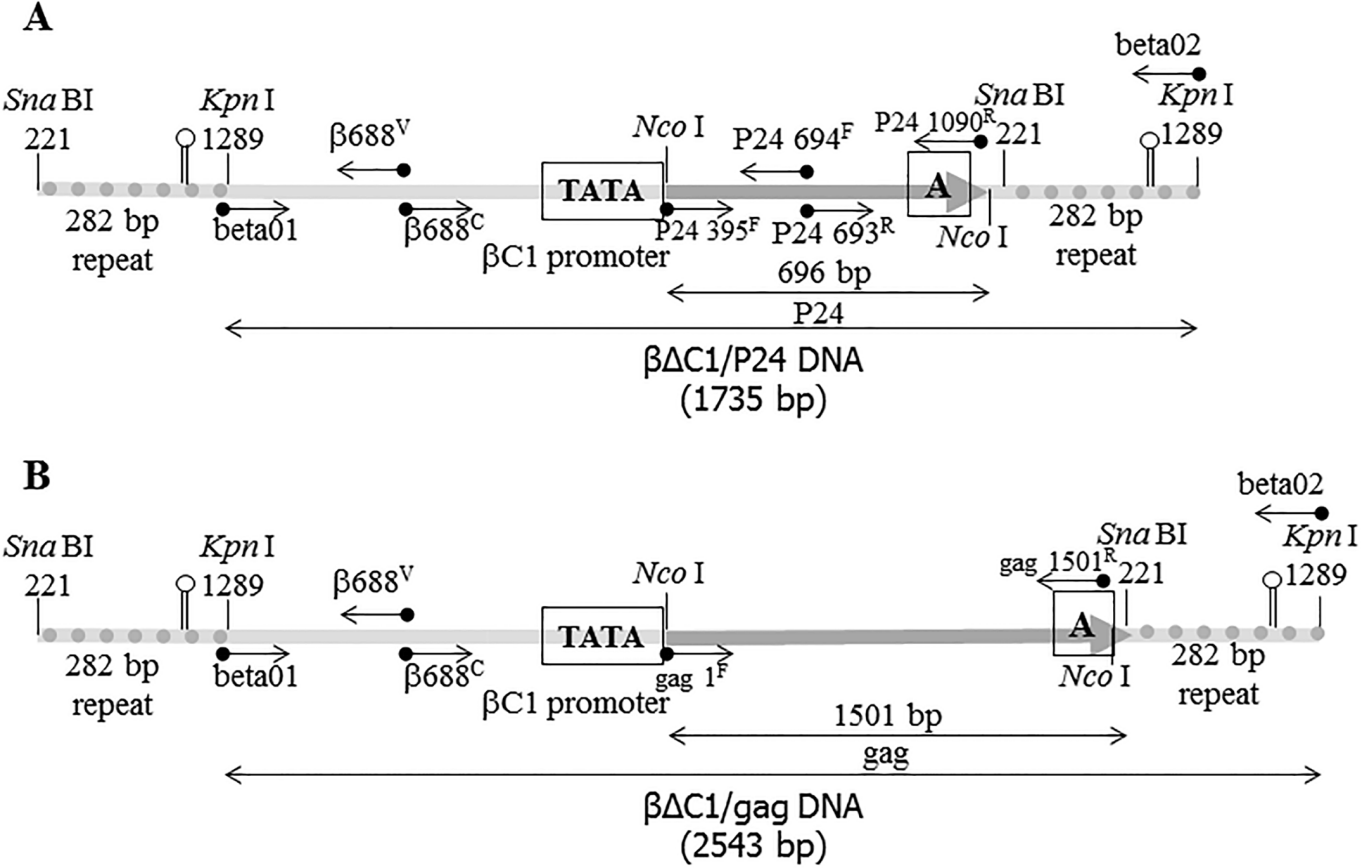
The schematic map of (A) pBinβΔC1/p24 and (B) pBinβΔC1/gag constructs. In (A) the CLCuMBΔC1 DNA with a direct repeat of 282 bp flanked the 696 bp DNA fragment of HIV-1 p24 and in (B) flanked the 1501 bp DNA fragment of HIV-1 gag. The direct repeat of the CLCuMB DNA is represented by the dotted rods. The restriction enzymes used in the construction of the cassettes and their nucleotide positions in CLCuMB DNA (Acc. No. AJ298903) are shown by the vertical bars. The positions of the polyadenylation signal and the TATA box in βC1 promoter, upstream of the initiation codon of the βC1 ORF are shown by the A and TATA signals on the diagram, respectively. The one sided arrows are used to show the position of the primers used in the construction of the constructs and further analysis of the resulted DNAs in inoculated plants. The two sided arrows are used to show the expected size of the amplified fragments. These constructs cloned separately as *Kpn*I/*Sna*BI fragments into the pBin20 binary vector were used in agroinoculation experiments.

To assay the possibility of using CLCuMB as an HIV-1 coat protein (CP) gene expression vector, two recombinant constructs were produced by the insertion of *p24* and *gag* genes of HIV-1 into pBin-1.2βΔC1 to generate pBinβΔC1/p24 and pBinβΔC1/gag, respectively. To do so, the recombinant CLCuMB/p24 and CLCuMB/gag constructs were first produced by insertion of a 696 bp PCR product of p24 and a 1501 bp PCR product of gag into the *Nco*I site of pTZβΔC1 (Fig 1) to generate pTZβΔC1/p24 and pTZβΔC1/gag, respectively. These PCR products were produced using a HIV-1 cDNA clone (pCMVR8.74, Addgene; https://www.addgene.org/22036), *Platnium Taq* DNA Polymerase High Fidelity (Invitrogen) and oligonucleotide primer pairs p24395^V^/1090^C^ and gag1^V^/1501^C^ (Table 1), each containing an *Nco*I site at its 5′ end, for p24 and gag genes amplification, respectively. The 1735 bp βΔC/p24 and 2500 bp βΔC/gag were released from pTZβΔC/p24 and pTZβΔC/gag via digestion with *Kpn*I and each DNA substituted with the full length CLCuMB DNA within the pBin-1.2β already digested with *Kpn*I to make the pBinβΔC1/p24 and pBinβΔC1/gag constructs, respectively, in which the CLCuMBΔC1 DNA with a direct repeat of 282 bp flanked the 696 and 1501 bp fragments of p24 and gag, respectively (Fig 1). Proper orientation of the inserted sequences into pBinβΔC1/p24 and pBinβΔC1/gag constructs was checked by restriction enzyme analysis using *Pst*I and *Kpn*I endonuclease enzymes and PCR using different primer pairs (data not shown). The pBinβΔC1/p24 and pBinβΔC1/gag constructs were separately introduced into *Agrobacterium tumefaciens* strain C58 by electroporation.

**Table 1:** List, sequences and other details of oligonucleotide primers used in this study.

Primers*	Size (nt)	Nucleotide position	Sequences from 5' to 3'	URS
gag 1^F^	31	1–24	TTCCATGGGGTGCGAGAGCGTC AGTATTAAG	*Nco*I
gag 1501^R^	31	1479–1501	TTCCATGGTTATTGTGACGAGGG GTCGCTGC	*Nco*I
p24 395^F^	30	395–416	TTCCATGGCCTATAGTGCAGAACA TCCAGG	*Nco*I
p24 1090^R^	27	1072–1090	TTCCATGGTTACAAAACTCTTGCTTTA	*Nco*I
p24 694^F^	19	694–712	GGGAAGTGACATAGCAGGA	
p24 693^R^	19	675–693	CTTGGTTCTCTCATCTGGC	
p24 626^F^	18	626–643	GCAGAATGGGATAGAGTG	
p24 767^R^	20	748–767	GGATAGGTGGATTATGTGTC	
Beta01 (Beta 1285^V^)	25	1285–1309	GGTACCACTACGCTACGCAGCAGCC	*Kpn* I
Beta02 (Beta 1290^C^)	25	1266–1290	GGTACCTACCCTCCCAGGGGTACAC	*Kpn* I
Beta 688^c^	22	667–688	GTCTCGATCATCACTTTATCCC	
Beta 689^v^	25	689–712	GTATTACACGTGTTGTCATGTTTGG	
TYLCV-[Ab] 349^V^	20	349–368	CTCGAAGGTTCGCCGAAGGC	
TYLCV-[Ab] 1997^C^	22	1976–1997	CGCGGCCATGGAGACCTAATAG	
Ef1^F^ (tobacco)	24		GGTTAAGATGATTCCCACCAAGCC	
Ef1^R^ (tobacco)	24		GACAACAACAACAGCAACAGTCTG	

* Beta: Cotton leaf curl Multan betasatellite, CLCuMB (AJ298903); TYLCV-[Ab]: Iranian isolate of Tomato yellow leaf curl Virus (FJ353946)

^V^ Virion-sense strand

^C^ Complementary-sense strand

^F^ Forward strand

^R^ Reverse strand

URS: Underlined Restriction Site

Ef: elongation factor

### Infectivity assays

*A*. *tumefaciens* cultures harboring a tandem repeat of either the wild-type or the recombinant CLCuMB/p24 and CLCuMB gag constructs or an infectious construct of TYLCV-[Ab] as the helper virus were grown separately at 28°C for 36 to 48 h. Wild-type or each recombinant CLCuMB culture was diluted to a concentration of optical density of 0.1 at 600 nm and mixed in equal proportions with the helper viral culture having the same concentration. *N*. *glutinosa* plants, at the four-leaf stage, were inoculated by injection of 100 μl of each mixed culture into the axillary buds and several leaf nodes of the decapitated main stem of each plant using a 100 μl Hamilton syringe. Newly-emerged leaves were sampled 21 days post-inoculation (dpi) and total DNA was extracted from 200 mg leaf tissue of each plant using cethyl-trimethyl-ammonium bromide (CTAB) as described by Gawel and Jarret [42]. The presence of helper viral and CLCuMB DNAs were detected in total DNA extracts by PCR analysis as previously described [28], using the virus, CLCuMB, p24 and gag specific primers as outlined in Table 1.

### Reverse transcription (RT)-PCR

Total RNA was extracted from from newly-emerged leaves of plants inoculated with either wild-type or recombinant CLCuMB in the presence of TYLCV-[Ab] as the helper virus using the RNX-Plus solution (Cinnagen, Iran). The extracted RNAs treated with RNase-free DNase (Fermentas). Total RNA extracted from leaves of a healthy plant was used as negative control. One μg of DNase treated RNA of each sample was used for first-strand cDNA synthesis in a 20 μl reaction containing 200 U M-MuLV reverse transcriptase, 100 pmol oligo-dT primer (18-mer), 15 pmol dNTPs and 20 U RNase inhibitor (all from Fermentas) in a 20 μl final volume. A pair of p24 specific primers (p24-626^V^/767^C^, Table 1) was used to amplify a 142 bp fragment (cDNA) of *p24* transcript. A tobacco elongation factor 1-alpha (Ef1-alpha, Acc. No. NM_001326165.1) primer pair (Ef1^F^ and Ef1^R^, Table 1) was used to amplify a 112 bp fragment of the tobacco Ef1-alpha transcript as an internal control.

### Quantitative Real-time PCR analysis

Quantitative Real-time PCR (qRT-PCR) was performed to quantify the transcription level of p24 in *N*. *glutinosa* plants co-inoculated with DNAβΔC1+p24 and TYLCV-[Ab] infectious constructs. qRT-PCR reactions were carried out in a real time thermal cycler (Bioer, China) in 20 μl reaction mixtures, each containing 1 μl cDNA (template), 25 μM of each primer (p24 626^F^/p24 767^R^, Table 1), 1 × Syber Green buffer and 0.12 units of *Taq* DNA polymerase (Bioer, China). The mixture was denatured for 5 min at 95°C and subjected to a 40 cycle-amplification program of 94°C for 10 s, 55°C annealing for 15 s and 72°C of extension for 30 s. The specificity of the amplicons was evaluated based on melting curves resulted by heating the amplicons from 54 to 95°C. Amplification reactions were carried out on cDNA generated from total RNA extracted from newly-emerged leaves harvested 21 dpi from four *N*. *glutinosa* plants co-inoculated with the pBin1.2βΔC1/p24 construct and TYLCV-[Ab] as the helper virus. The threshold cycle (*CT*) for each sample was calculated using Linegene K software (Bioer, China) based on the method of Larionov et al. [43]. In qRT-PCR data analysis, absolute quantification of *p24* transcripts was done based on a standard curve [44], which was drawn by plotting the *CT* against the known concentrations of the p24 control templates. The purified pBinβΔC1/p24 DNA containing cloned *p24* gene sequence was used as the p24 control template. The plasmid DNA concentration was quantified by spectrophotometry using a NanoDrop ND-1000 spectrophotometer (NanoDrop Technologies). A series of dilutions of plasmid DNA in quantities ranging from 4 to 100 ng/μl was prepared and used as the qRT-PCR reaction template to generate the standard curve. Calculation of the p24 cDNA concentration in plants co-inoculated with the pBin1.2βΔC1/p24 and TYLCV-[Ab] as the helper virus was performed according to the standard curve.

### Enzyme-linked immunosorbent assay (ELISA)

To investigate expression of *p24* in inoculated plants, ELISA test was performed using HIV-1 Ab&Ag Fourth generation Enzyme Immunoassay kit (Dia-Pro Company). This kit is usually used to determine HIV-1 type 1&2&O and *p24* HIV-1 Antigen in human serum and plasma. In this study, the kit was used to detect the *p24* protein in *N*. *glutinosa* and *N*. *benthamiana* plants co-inoculated with DNAβΔC1+p24 and TYLCV-[Ab] infectious constructs containing the replicative forms of DNAβΔC1+p24 as determined by PCR analysis. Non-inoculated plants and plants inoculated with the helper virus (TYLCV-[Ab]) alone or with the wild type CLCuMB in the presence of TYLCV-[Ab] was used as control plants, negative for the presence of *p24* protein.

Fresh leaf tissues (100 mg) were ground in liquid nitrogen and homogenized in 200 μl of 50 mM potassium phosphate buffer (pH 7). The homogenate was incubated on ice for 30 min and centrifuged at 4°C for 5 min at 12000 rpm. One hundred μl of aqueous phase of each sample was used for coating microtiter plates. The test was conducted according to the manufacture’s protocol. Finally, the reaction of HIV-1 Ab to *p24* protein was assayed by measuring the optical density (OD) of protein samples at 405 nm using an ELISA reader (Bio-Tek, Multi-Mode Microlpate Reader). Plant protein samples with OD higher than average + 3×SD of ODs of control plants were evaluated as positive samples.

The positive antigen (Ag) control of the ELISA kit containing non-infectious recombinant p24 Ag with a concentration of 100 pgml^-1^ was also used in ELISA test to estimate the p24 concentration in plant extracts. To do so, the average OD of healthy plant extracts (negative control) was subtracted from the OD of corresponding experimental samples and the products were used in comparison with positive control.

### Statistical analyses

Statistical analyses was performed in data, the mean values of the replicate samples were calculated, an analysis of variance (ANOVA) was employed to investigate significant differences. Duncan’s multiple range tests in MINITAB 16 (Minitab, Inc., Pennsylvania, USA) software were used to carry out the mean comparisons. In all cases, a *P* value of 0.05 was considered significant.

## Results

### Replication of βΔC1/p24 construct in plants in the presence of TYLCV- [Ab] as the helper virus

**Fig 2:**
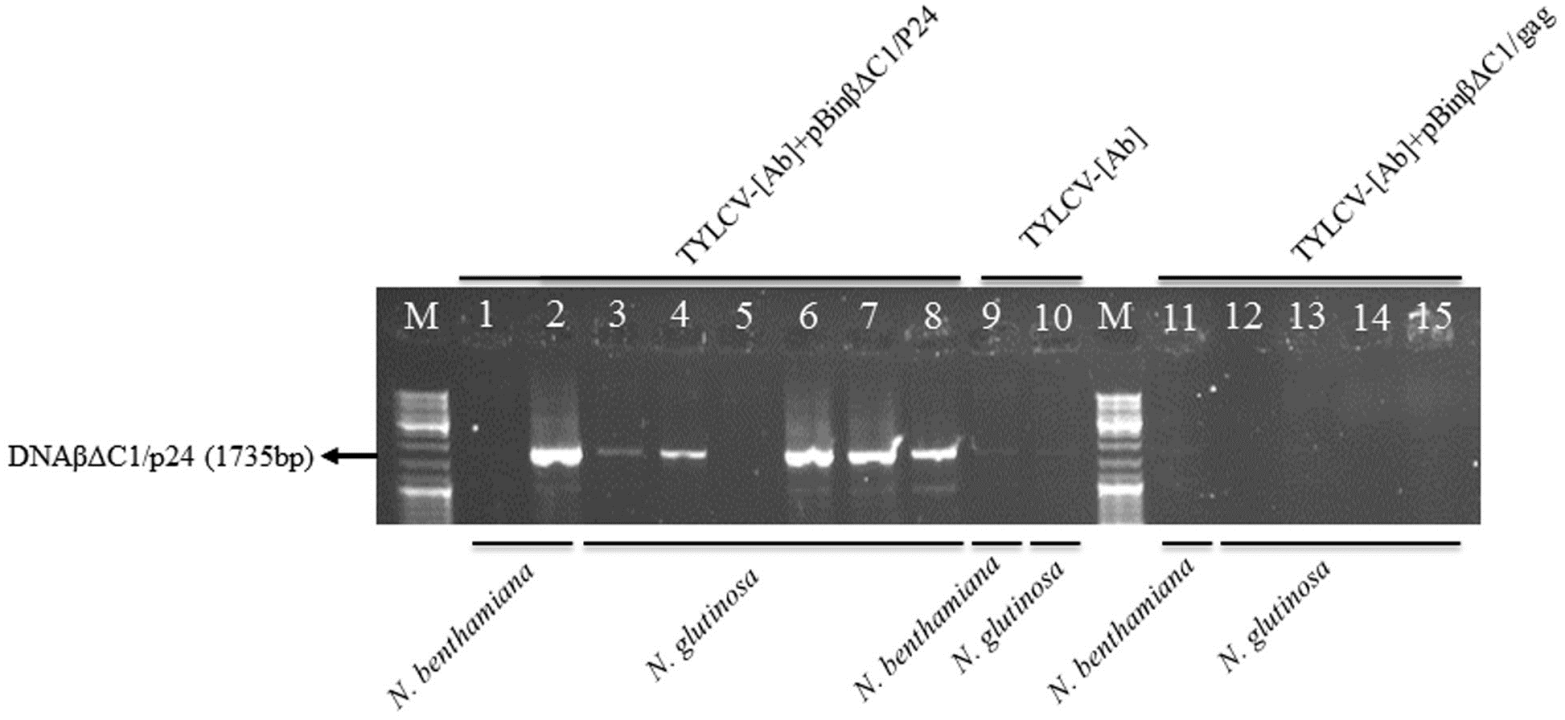
Transreplicon of recombinant βΔC1/p24 DNA (1735 bp) in *N*. *glutinosa* and *N*. *benthamiana* plants. The DNA fragments were obtained from plants, as indicated at the bottom of the lanes, co-agroinoculated with the pBinβΔC1/p24 construct and the helper virus TYLCV-[Ab] (lanes 1–8) by PCR using specific adjacent beta1285^V^/1290^C^ primer pair (Table 1). No DNA was amplified from the extracts of plants inoculated with the helper virus alone (lanes 9–10) and plants co-inoculated with the pBinβΔC1/gag construct and the helper virus (lanes 11–15) using the same primer pair. M, marker DNA ladder (Fermentas).

The integrity of the 696 bp DNA fragment of HIV-1 p24 and the 1501 bp DNA fragment of HIV-1 gag inserted into pTZβΔC/p24 and pTZβΔC/gag constructs, respectively, was confirmed by sequence analysis. The pBinβΔC1/p24 and pBinβΔC1-gag constructs generated from pTZβΔC/p24 and pTZβΔC/gag constructs, respectively, were separately agroinoculated into *N*. *glutinosa* and *N*. *benthamiana* seedlings in the presence of TYLCV-[Ab] as the helper virus. Some plants were also inoculated with the helper virus alone and the wild type CLCuMB construct in the presence of the helper virus. Specific primer pairs for both the CLCuMB and the helper virus were used in PCR to detect recombinant CLCuMB and the viral DNAs in systemically infected tissues of agroinoculated plants 21 dpi. A DNA band of the expected size of the TYLCV-[Ab] genome was detected in all inoculated plants by PCR using a specific TYLCV-[Ab] primer pair (Table 1) (data not shown) demonstrating successful systemic infection of helper virus in all plants tested. The recombinant full-length βΔC1/p24 CLCuMB DNA (1735 bp) was amplified from the DNA extracts of most (5 out of 6) *N*. *glutinosa* plants (Fig 2, lanes 3–8) and one out of two *N*. *benthamiana* plants (Fig 2, lanes 1–2) co-inoculated with the pBinβΔC1/p24 construct and the helper virus construct when two specific adjacent primers of CLCuMB (beta1285^V^/1290^C^, Table 1) were used in PCR. However, no DNA was amplified from the DNA extracts of plants co-inoculated with the helper virus alone (Fig 2, lanes 9–10) and the pBinβΔC1/gag construct in the presence of the helper virus (Fig 2, lanes 11–15).

**Fig 3:**
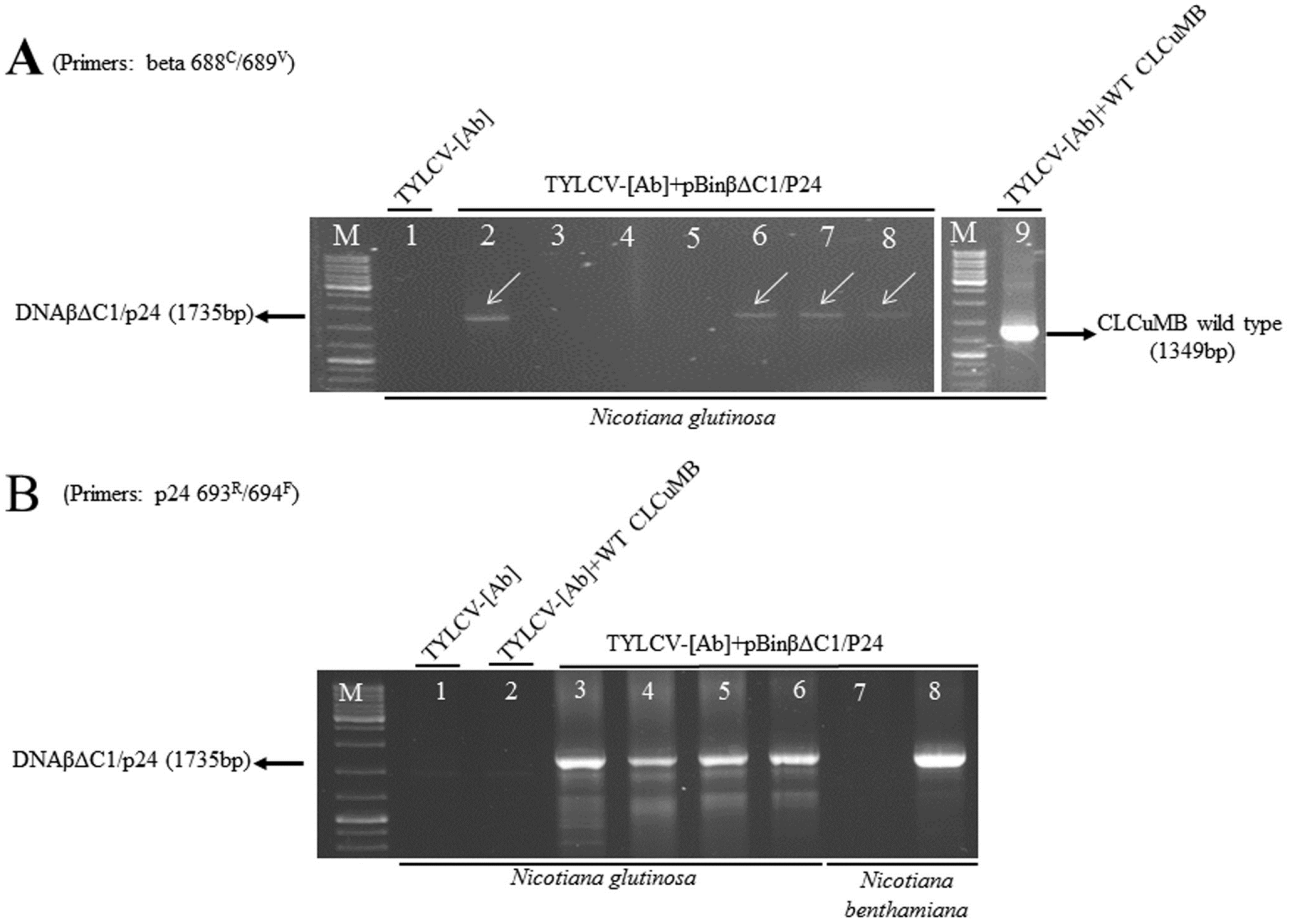
Transreplicon of recombinant βΔC1/p24 DNA (1735 bp) in *N*. *glutinosa* and *N*. *benthamiana* plants. DNA fragments were amplified from plants, as indicated at the bottom of the lanes, co-agroinoculated with pBinβΔC1/p24 construct and the helper virus TYLCV-[Ab] (A, lanes 1–8 and B, all lanes) by PCR using specific adjacent beta 688^C^/689^V^ (A) and p24 693^R^/694^F^ (B) primer pairs. Transreplicon of the wild-type (WT) CLCuMB DNA (1349 bp) in an *N*. *glutinosa* plant co-agroinoculated with WT CLCuMB construct and the helper virus TYLCV-[Ab] is shown in A lane 9. No DNA was amplified from the DNA extracts of *N*. *glutinosa* plants inoculated with the helper virus alone (A and B lanes 1) and a plant co-inculated with the WT CLCuMB construct and the helper virus (B lane 2) when the p24 693^R^/694^F^ primer pair was used. M, marker DNA ladder (Fermentas).

Upon infection of plants cells with the virus, the recombinant circular βΔC1/p24 molecule as a replicating molecule is released from the linear βΔC1/p24 cassette during replication process and subsequently replicated as circular dsDNA molecules through rolling-circle replication. The beta1285^V^/1290^C^ primer pair is capable of amplifying the βΔC1/p24 DNA fragment from both the transformed linear βΔC1/p24 cassette and the recombinant circular βΔC1/p24 molecule and therefore the nature of amplified fragment could not be determined by using this primer pair. To verify that the circular replicative forms of the recombinant βΔC1/p24 DNA were produced in inoculated plants and served as template for PCR amplification, other PCR experiments were performed in which two pairs of specific adjacent primers, beta 688^C^/689^V^ and p24 693^R^/694^F^ (Table 1), designed from DNA β and p24 regions of βΔC1/p24 DNA (Fig 1), respectively, were used. These primers are capable of amplifying the 1735 bp βΔC1/p24 DNA fragment only from the recombinant circular βΔC1/p24 molecule, but not from the transformed linear βΔC1/p24 DNA cassette. Four plants out of seven *N*. *glutinosa* plants co-inoculated with βΔC1/p24 and TYLCV were found to contain PCR products approximating the size predicted for the complete 1735 bp circular βΔC1/p24 molecule (Fig 3A, lanes 2–8) when the beta 688^C^/689^V^ specific adjacent primer pair were used. The same circular βΔC1/p24 fragments were amplified from the extracts of the four aforementioned *N*. *glutinosa* plants (Fig 3B, 3–6) and one plant out of two *N*. *benthamiana* plants (Fig 3B, 7–8) co-inoculated with βΔC1/p24 and TYLCV when the p24 693^R^/694^F^ specific adjacent primer pair were used in PCR reaction. These observations indicated that the recombinant βΔC1/p24 construct was successfully replicated in the presence of helper TYLCV in *N*. *glutinosa* and *N*. *benthamiana* plants and that βΔC1 could be used as a HIV-1 *p24* gene delivery vector to plants. However, the replacement of βC1 gene with larger foreign DNA fragment, i.e. HIV-1 *gag* gene, cannot be tolerated.

### Detection of *p24* transcripts in plants co-inoculated with βΔC1/p24 construct and the helper virus

**Fig 4:**
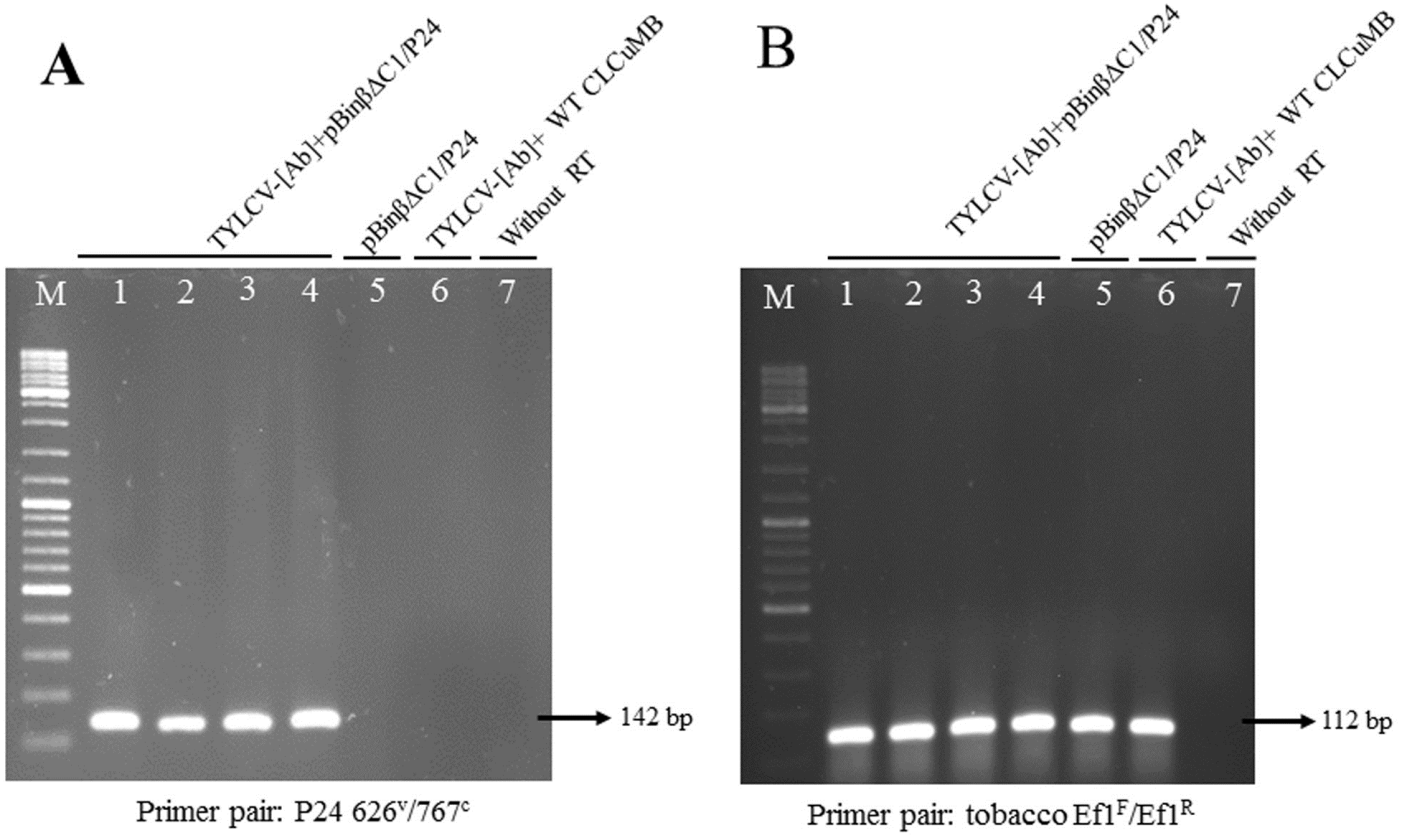
Amplification of the 142 bp p24 DNA fragments (A) and 112 bp tobacco elongation factor (Ef) 1-alpha DNA fragments (B). The DNA fragments were obtained by RT-PCR using the total RNA extracted from *N*. *glutinosa* plants inoculated with the constructs as shown at the top of the lanes using a pair of either p24 (A) or tobacco EF1 (B) specific primer pairs as shown at the bottom of each panel. The p24 142 bp DNA fragment and 112 bp tobacco EF1 DNA fragment were absent (lane 7 of each panel) in PCR control reactions without RT from the extracted RNA of *N*. *glutinosa* plants co-inculated with βΔC1/p24 construct in the presence of TYLCV-[Ab].

To analyze the presence of *p24* transcripts in inoculated plants, RT-PCR was performed. Use of a p24-specific primer pair (p24 626^V^/767^C^, Table 1) yielded a specific DNA band of approximately 142 bp in size (Fig 4A, lanes 1–4) from the RNA extracts of *N*. *glutinosa* plants co-inoculated with recombinant βΔC1/p24 construct and the helper virus. This DNA product was absent in RT-PCR reactions from *N*. *glutinosa* plants inoculated with the βΔC1/p24 construct alone (Fig 4A, lane 5) or co-inoculated with the wild type CLCuMB and TYLCV-[Ab] (Fig 4A, lane 6) and also in PCR control reactions (without RT) of total RNA extracted from *N*. *glutinosa* plants co-inoculated with βΔC1/p24 construct in the presence of TYLCV-[Ab] as the helper virus (Fig 4A, lane 7). Under the same conditions, the level of DNA products amplified from the tobacco *EF 1-alpha* transcripts (subsequently used as an internal control in real time PCR test) was similar in the same plants tested (Fig 4B). These data demonstrate that *p24* transcripts were only present in plants co-inoculated with recombinant βΔC1/p24 construct in the presence of the helper virus.

### Quantification of *p24* transcripts

Quantitive real time PCR (qRT-PCR) was used to estimate the transcription concentration of *p24* in *N*. *glutinosa* plants co-inoculated with the pBin1.2βΔC1/p24 and TYLCV-[Ab] constructs. These plants have been already proven to have the circular replicative forms of the recombinant βΔC1/p24 DNA. qRT-PCR was performed on cDNA generated from total RNA extracted from newly-emerged leaves harvested 21 dpi from the four aforementioned *N*. *glutinosa* plants. The average concentration of *p24* cDNAs generated from these plants was calculated to be 38 ngml^-1^ according to a standard curve, which was prepared by plotting the *CT* of the p24 control templates against their known concentrations. These results confirmed that the *p24* was transcribed in a relatively high level in plants infected with pBin1.2βΔC1/p24 construct in the presence of the helper virus.

### *p24* protein produced in plants co-inoculated with βΔC1/p24 construct and TYLCV-[Ab] as the helper virus

**Fig 5:**
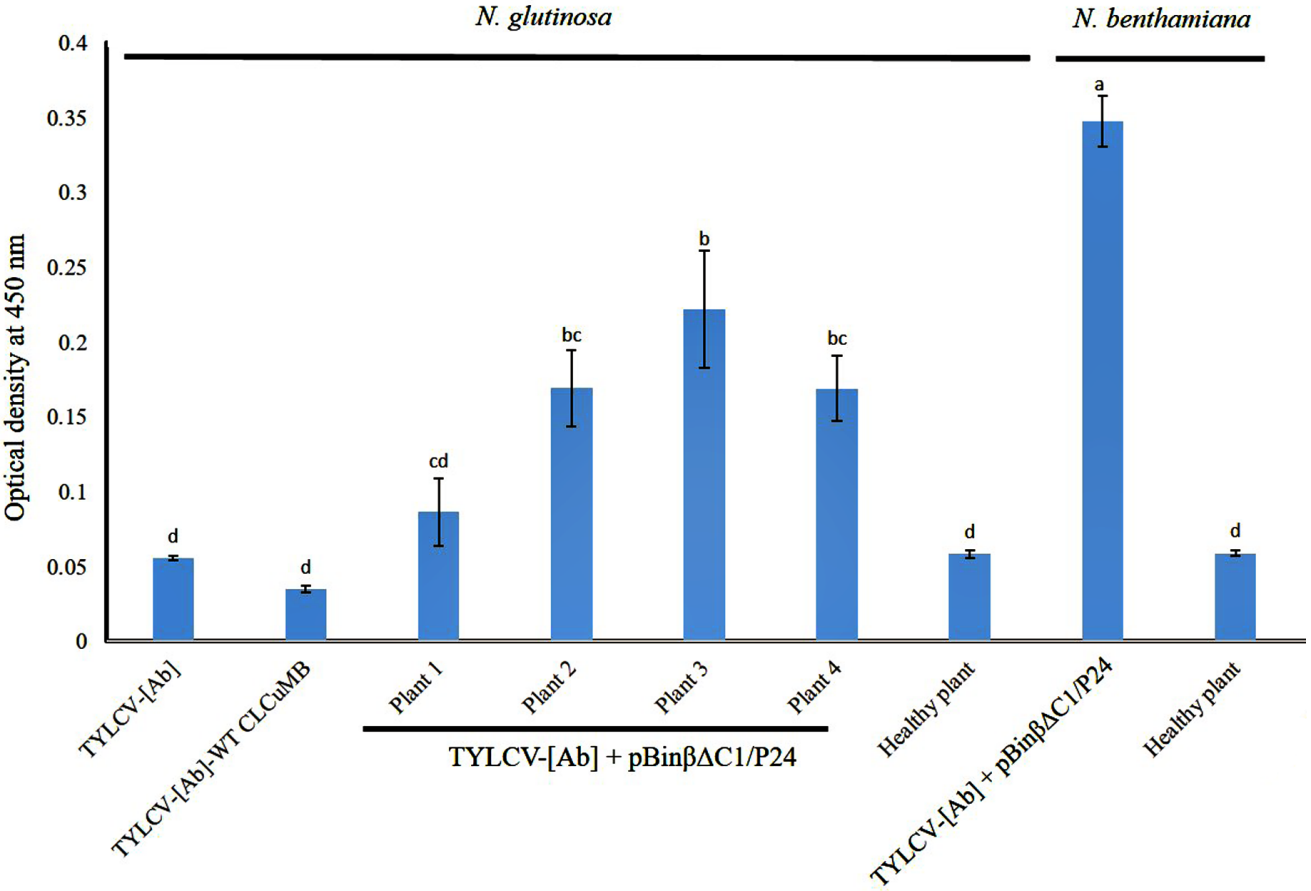
Optical density values at 405 nm of the ELISA plate readings of protein samples from inoculated plants. Plants, as indicated at the top of the columns, inoculated with the constructs as indicated at the bottom of the columns. The value represented for each treatment is the mean of three replicates. Bars with different letter are significantly (*p* ≤ 0.05) different. Quantitation of HIV-1 p24 protein in plant extracts was estimated by ELISA using the positive antigen (Ag) control of the ELISA kit with a definite (100 pgml-1) concentration. The p24 protein concentration was estimated to be 407.1 ngml^-1^ in a *N*. *bethamiana* plant extract (corresponding to a yield of 814.2 ng per g fresh plant tissue) whereas the concentration of the same protein in four *N*. *glutinosa* plant extracts ranged from 39.4 to 231.3 ngml^-1^ (corresponding to a yield of 78.8 to 462.6 ng per g fresh plant tissue). This showed that *N*. *bethamiana* was a more suitable host for production of HIV-1 *p24* protein using the CLCuMB-based expression vector.

To indicate the presence of *p24* protein in inoculated plants, protein extracts (without concentration or purification) were prepared from leaves of four *N*. *glutinosa* plants and one *N*. *benthamiana* plant co-inoculated with pBin1.2βΔC1/p24 and TYLCV-[Ab] constructs and positive for *p24* transcripts as shown in RT-PCR experiment and absolute real time PCR. Protein extracts of these plants were subjected to ELISA test using a p24 antibody. Extracts from healthy *N*. *glutinosa* and *N*. *benthamiana* plants and *N*. *glutinosa* plants inoculated with the TYLCV-[Ab] alone or with the wild type CLCuMB in the presence of TYLCV-[Ab] were used as control plants. Optical density (OD) values at 405 nm of the ELISA readings of protein samples from plants co-inoculated with pBin1.2βΔC1/p24 and TYLCV-[Ab] constructs were compared with those from healthy plants and from plants inoculated with the TYLCV-[Ab] alone or with the wild type CLCuMB in the presence of TYLCV-[Ab] (Fig 5). This comparison indicated that the average OD of protein samples (3 repeats for each treatment) of all four *N*. *glutinosa* plants and one *N*. *benthamiana* plant co-inoculated with pBin1.2βΔC1/p24 and TYLCV-[Ab] constructs were significantly higher (*p* ≤ 0.05) than the average OD + 3×standard deviation of protein samples of healthy plants. No significant difference (*p* ≤ 0.05) was observed between the OD values from the healthy *N*. *glutinosa* plant samples and those from *N*. *glutinosa* plants inoculated with the TYLCV-[Ab] alone or with the wild type CLCuMB in the presence of TYLCV-[Ab]. These results indicated that *p24* protein was produced in plants co-inoculated with pBin1.2βΔC1/p24 and TYLCV-[Ab] constructs.

## Discussion

Plant-made recombinant proteins can be produced using either stably transformed plants or by transient expression [45, 46]. Plant viral vectors are widely used for transient expression in plants [7, 47, 48]. In this study a transient expression approach with an infectious plant virus-derived vector was used to make recombinant HIV-1 coat protein in plants. The transient expression approach has several advantages over production from stably transformed plants; in particular, transient expression is very fast, so that in the current study the production of *p24* transcripts and protein in systemically infected tissues of agroinoculated plants was completed in 21 dpi. Whereas it usually takes several months to produce stably transformed plants [40, 49]. Furthermore, due to varying transgene copy number and/or their position effects in most stable transformation systems, these systems may suffer from unpredictable levels of transgene expression as a result of gene silencing [50].

The first plant viruses to be proposed as potential gene vectors were DNA viruses [51, 52], because at the time they were the only viruses for which genetic manipulation was possible. Thus, viruses from two families, the *Caulimoviridae* (circular double-stranded DNA genomes) and the G*eminiviridae* (circular single-stranded DNA genomes), were extensively investigated as potential vectors in the 1980s [53]. This was followed by examination of various designs for amplification and expression of foreign proteins in plants by geminiviral replicon gene vectors [11, 12, 54–58]. The advantages of geminiviruses over plant RNA virus expression vectors have been described. It has been reported that the geminivirus expression vectors represent a cutting edge method in the generation of vaccines and other therapeutic proteins in plants [59]. However, Tobacco mosaic virus-based delivery and expression systems have also been used extensively in scales needed for commercial vaccine production [60–62].

Several expression systems based on geminiviruses engineered for vaccine production including HIV-1 type C *p24* [11] have been recently introduced [59]. On this basis, many vaccine and therapeutic proteins have been expressed in plants using geminivirus-based vectors [59]. However, geminiviruses-based vectors like other deliver and expression vectors have also some disadvantages. The entire genome of geminiviruses possess overlapping ORFs as well as the viral regulatory elements involved in their replication and gene expression [63]. These characteristics may be disadvantageous for their applications as gene delivery systems. In addition, there are size constraints on the viral DNA for replication [64] and geminiviral genomes carrying large foreign DNAs are usually unstable and produce smaller progeny molecules [28, 64–66]. However, it has been reported that efficient replication of recombinant geminiviral vectors carrying long inserts can be achieved using transient expression methods like agroinfiltration within 3–5 days [67, 68]. The finding that the CLCuMB which lacks overlapping ORFs can be used as a plant and animal gene delivery and expression vector to plants [10, 29] instigated this study to transfer a part of HIV-1 coat protein gene, i.e., *p24* to plants for production of *p24* mRNAs and subsequent production of *p24* protein in the host plants.

In addition to a geminivirus-based vector advantages, the CLCuMB-based expression vector offer considerable advantages for delivery and expression of foreign genes in plants including the HIV-1 coat protein gene. Kharazmi et al. [10] discussed the advantages of a CLCuMBΔβC1 as a virus induced gene silencing vector. CLCuMB has almost no sequence homology to its helper geminiviruses on which it depends for its replication, encapsidation, insect transmission and systemic movement in plants [25, 27]. Therefore, inserting a foreign DNA fragment in CLCuMB avoided homologous gene silencing following infection by helper viruses. It has been shown that the βC1 ORF does not contain elements essential for CLCuMB replication by the helper virus, thus it is possible to delete the βC1 ORF but retain its promoter and termination signal without having considerable effect on CLCuMB replication [10, 28]. Since CLCuMB-based expression vector introduced in this study has its own promoter and termination signal, there is no need to add a promoter and terminator that may result in its instability due to increasing its size. The βC1 protein of CLCuMB is an essential pathogenicity determinant [26] and functions as a suppressor of RNA silencing [69]. Therefore, replacing the βC1 gene with an external DNA fragment similar to that used in this study, eliminates the vector’s ability to induce severe disease symptoms in the presence of its cognate helper virus i.e., Cotton leaf curl virus, and most other helper viruses, which would otherwise mask the possible expression of the introduced gene phenotype.

The results of RT-PCR and qRT-PCR in this study indicated that the *p24* transcript was produced in inoculated plants with a concentration of 38 ngml^-1^. Injection of extract of these plants containing a large number of *p24* transcripts to an animal such as a rabbit may trigger gene silencing against new entrance of HIV-1 [70–72]. The results of ELISA test indicated that *p24* protein produced in *N*. *glutinosa* and *N*. *benthamiana* plants co-inoculated with DNAβΔC1/p24 and TYLCV-[Ab] infectious constructs. Plants capable of producing proteins are interesting tools for the construction of edible oral vaccines originating from foreign organisms [31, 73]. Although there is a great deal of concern about the use of edible oral vaccine in animal health, in some cases it does not stimulate the animal’s immune system, resulting in immunogenicity being low [4, 74]. Kharazmi et al. [10] indicated that CLCuMB replicates in tobacco, tomato and datura plants in the presence of diverse helper viruses. Co-replication of CLCuMB with different geminiviruses, especially the broad host range Beet curly top virus introduces it as a prospective gene delivery vector to a considerably large number of plant species hosting different geminiviruses that support the CLCuMB [10]. Since many edible hosts infected by the viruses support CLCuMB replication, it is possible to produce the *p24* protein in these edible hosts following inoculation with DNAβΔC1/p24 in the presence of a helper virus and then use these plants for the production of edible vaccines. The ability of geminivirus-based vectors to express HIV-1 coat protein in plants has been already reported [11, 75, 76]. It has been already reported that the *p24* capsid protein from HIV-1 subtype C was produced in *Arabidopsis thaliana* and *Daucus carota* using a protein expression construct having an endoplasmic reticulum-directing SEKDEL sequence [31]. These plants were able to induce systemic immune response in mice after oral delivery of fresh plant material [31]. Because the CLCuMB-based delivery and expression vector described in this study has its own promoter activity, it offers the possibility of production of animal recombinant proteins and their vaccines in plants. However, extended works should be done experimentally to confirm the ability of this delivery and expression system to produce vaccines.

The strategy used in this study was successful in achieving *p24* gene replication, transcription and expression in plants co-inoculated with pTZβΔC/p24 construct (harboring the *p24* gene with 700 bp in size) and TYLCV-[Ab] as the helper virus. However, plants inoculated with pTZβΔC/gag construct (harboring the *gag* gene with 1501 bp in size) in the presence of the same helper virus were not infected with the gag construct. These results demonstrated that CLCuMB could be used as a HIV-1 coat proteins delivery vector to plants, but that the size of the insert may be a limiting factor. It has been shown that the CLCuMBΔC1 DNA tolerates DNA insertions of up to 720 bp without significant effect on its replication [29]. HIV-1 p24 was in this rage. Furthermore, the foreign genes could be expressed by the CLCuMB promoter after delivery to a wide range of plants by CLCuMB-based vector system. However, attempts to insert larger DNA fragments (such as HIV-1 *gag* gene with 1501 nt), due to increasing the heterologous DNA sequences compared to betasatellite sequence in recombinant CLCuMB DNA, resulted in truncation of the fragment during replication, presumably by the helper virus replication-associated protein (Rep). Nevertheless, the results of this study demonstrated that CLCuMB could be used as a HIV-1 coat proteins delivery vector to plants, but that the size of the insert may be a limiting factor.

Although the CLCuMB-based vector used in this study failed to express the HIV-1 gag, the success of the system in expressing *p24* protein can lead to cheap production of this protein, which can be used in molecular farming circles. However, the yield of total p24 (79–814 ng per g fresh plant tissue) expressed by this system was very low compared to some other systems. For example, the yield of 1 μg p24 per g fresh weight in tobacco plant was reported by Meyers et al. [41]. Also Scotti et al. [77] obtained a yield of 400 μg per g plant tissue of an enveloped 100-nm virus-like particles (VLPs) containing complete HIV-1 gag protein. Considering these data, it can be assumed that a commercially viable expression system of recombinant proteins in plants should be at least in the yield rang of 100 ugg^-1^. The CLCuMB/p24 expression system as used under the circumstances of this study did not produce a commercially viable p24 yield. It is important to improve this expression system by new experiments using agroinfiltration-mediated transient expression method [49, 67, 68]. Furthermore, because since the onset of HIV vaccination, the *p24* protein has not been seriously touted as a vaccine antigen, in each expression system including the CLCuMB-based vector system the production of gag in the form of VLPs [37] should also be considered. One way to overcome the disadvantages of this system is to replace it with a non-infectious system. The latter can be made in such a way to express only viral Rep [4, 15]. Such a system can support the dependent replicon constructs which would be responsible for expression of desired proteins. Application of movement-components along with non-replicating Rep or application of Rep and movement protein-encoding constructs in Trans could also be considered [11, 78]. In these cases cell-to-cell movement is no longer necessary and this can create the potential to advance vector systems for expression of larger proteins such as gag. Furthermore, these constructs need to be introduced to plant cells by agroinfiltration rather than agroinjection. This makes possible assaying expression of the desired proteins over a shorter time (7–9 days) [78] compared to 21 days required by the experiments in the current study.
